# Unexpectedly Prolonged Serotonin Syndrome and Fatal Complications Following a Massive Overdose of Paroxetine Controlled-Release

**DOI:** 10.7759/cureus.50691

**Published:** 2023-12-17

**Authors:** Fumiya Inoue, Yuji Okazaki, Toshihisa Ichiba, Kenichiro Kashiwa, Akira Namera

**Affiliations:** 1 Emergency Medicine, Hiroshima City Hiroshima Citizens Hospital, Hiroshima, JPN; 2 Forensic Medicine, Hiroshima University, Hiroshima, JPN

**Keywords:** endoscopic decontamination, pharmacokinetics, serotonin syndrome, controlled-release formation, paroxetine

## Abstract

Symptoms caused by a selective serotonin reuptake inhibitor (SSRI) overdose are often mild and can be managed with supportive care and close monitoring, even when complicated by serotonin syndrome. There are limited pharmacokinetic data regarding massive overdoses of paroxetine, and the severity of an SSRI overdose is likely to be underestimated. We describe a fatal case of severe serotonin syndrome and acute respiratory distress syndrome (ARDS) following an overdose of controlled-release paroxetine. A 53-year-old male with depression presented with altered consciousness. He had ingested controlled-release paroxetine along with other medications. On arrival, he had ocular flutter and myoclonus, and blood examinations revealed acute kidney injury and rhabdomyolysis, which suggested serotonin syndrome. Computed tomography (CT) showed pharmacobezoars in the esophagus and stomach. Symptoms of serotonin syndrome and hypotension persisted despite administration of high doses of vasopressors with endotracheal intubation. We performed endoscopic decontamination to remove pharmacobezoars from the stomach. Finally, he developed severe ARDS and died due to respiratory failure on day 23. Sequential serum concentrations of paroxetine were 5.38 µg/mL at admission and 3.21 µg/mL on day 7, both above lethal levels. This case highlights the potential for fatal complications and prolonged toxicity in the case of a massive overdose of controlled-release paroxetine. We should recognize that such an overdose may be life-threatening and should consider aggressive interventions including endoscopic decontamination. A better understanding of the pharmacokinetics of a massive SSRI overdose would be helpful for optimal management.

## Introduction

Selective serotonin reuptake inhibitors (SSRIs) have been widely used as antidepressants because of their efficacy, tolerability, and safety. In cases of SSRI overdose, if ingested in small doses, the symptoms are usually mild and can be treated with supportive management and close monitoring even when complicated by mild serotonin syndrome [1,2]. The severity of the toxicity most commonly depends on co-ingestants and the dose of the drug taken. However, there is little pharmacokinetic information on the effects of overdoses of controlled-release formulations of paroxetine and co-ingestants, especially in patients who have ingested a massive dose (e.g., greater than 150 times the daily dose) [1,3,4]. It is essential to acknowledge that, in cases of a massive overdose, the normally predictable pharmacokinetic profile of drugs can be markedly altered, leading to unpredictable outcomes. We present a case of severe serotonin syndrome due to an overdose of the controlled-release formulation of paroxetine (Paxil CR®) in a patient with depression, which was eventually fatal due to acute respiratory distress syndrome (ARDS). To the best of our knowledge, the peak serum concentration of paroxetine in our patient is the highest among reported cases of paroxetine overdose, and this is the first case in which sequential serum concentrations revealed the pharmacokinetics of a fatal overdose of controlled-release paroxetine.

## Case presentation

**Figure 1:**
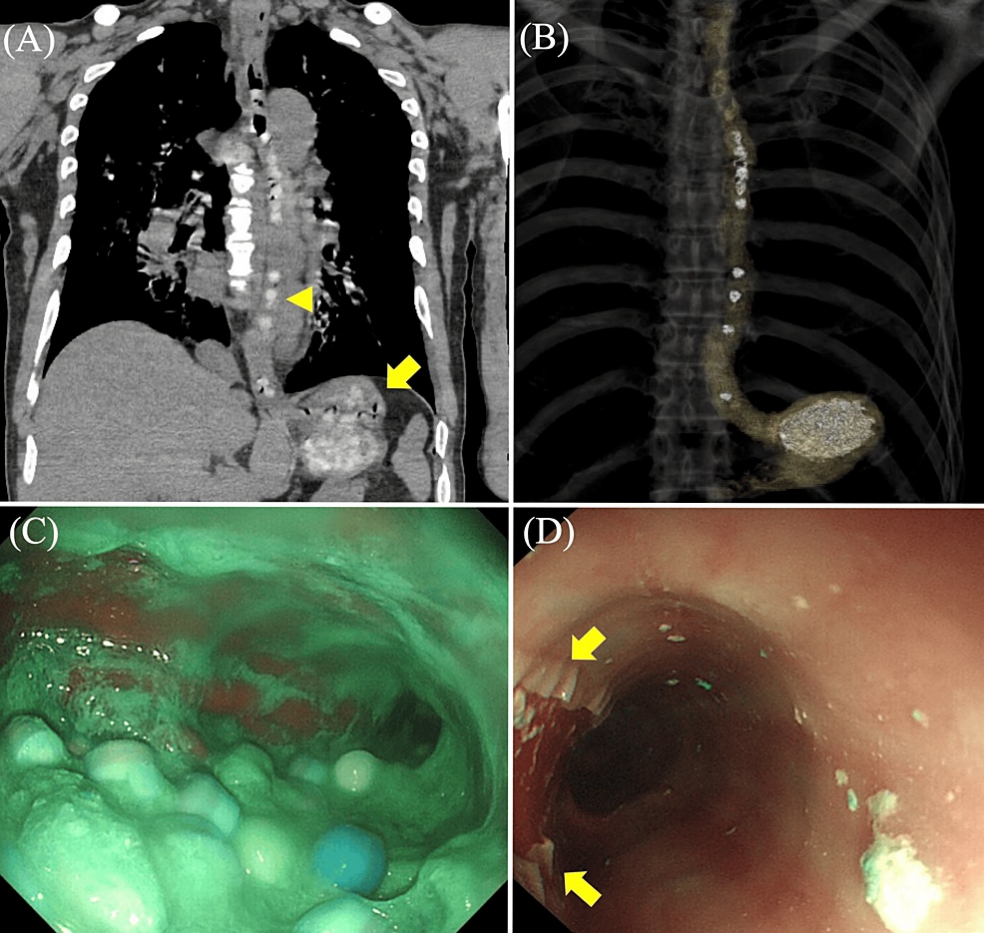
CT and EGD (A) Axial plain CT of the chest showed high attenuation areas in the esophagus (yellow arrowhead) and stomach (yellow arrow). (B) Three-dimensional volume-rendered CT showed the presence of ingested tablets in the esophagus and stomach. (C) EGD showed a large amount of pharmacobezoars in the stomach. (D) During endoscopic decontamination with a collection net, an esophageal tear occurred due to a collection net (yellow arrow). CT, computed tomography; EGD, esophagogastroduodenoscopy

A 53-year-old male with depression presented with an altered level of consciousness at our emergency department (ED). The last known-well time was approximately 24 hours. When he was found, empty press-through-package sheets of approximately 250 pills from his routine prescribed medication were scattered around him. His daily medication consisted of paroxetine controlled-release 25 mg, quetiapine 100 mg, trazodone 50 mg, aripiprazole 3 mg, flunitrazepam 4 mg, and zopiclone 2 mg. On arrival, his initial vital signs were as follows: GCS, 9 (E4V1M4); heart rate, 76 beats/minute; blood pressure, 104/73 mm Hg; oxygen saturation, 88% on 10 L/min supplemental O_2_; and temperature, 37.4°C. Physical examination revealed dilated pupils of 6 mm with absent light reflexes, ocular flutter, and spontaneous myoclonus of the extremities. Arterial blood gas revealed severe hypoxemia and lactic acidosis. Blood examination showed a markedly elevated creatine kinase level, severe acute kidney injury, and elevated C-reactive protein (Table 1). An electrocardiogram (ECG) showed that the QTc interval corrected by Fridericia formula was 0.606 sec. Whole-body computed tomography (CT) revealed aspiration pneumonia in the right lower lobe and pharmacobezoars in the esophagus and stomach (Figure 1A and Figure 1B). Although we did not know when he took the drugs and how many tablets he took, based on the presence of pharmacobezoars in the stomach, we performed endotracheal intubation and attempted to insert a nasogastric tube for gastrointestinal decontamination one hour after admission to the ED. However, the tube could not pass through the esophagogastric junction due to the occupied pharmacobezoars. During the two-hour stay in the ED, we administered 1 g of magnesium and 100 mL of 7% sodium bicarbonate intravenously for QT prolongation.

**Table 1:** Laboratory examination ALT, alanine transaminase; AST, aspartate transferase; γ-GTP, γ-glutamyl transpeptidase; ALP, alkaline phosphatase; LD, lactate dehydrogenase

	Hospital admission	Reference range
Arterial pH	7.28	7.31-7.41
Arterial pO_2_	67.2	80-100 mmHg
Arterial pCO_2_	43.6	35-45 mmHg
Arterial lactate acid	4.3	0.5-1.6 mmol/L
Arterial HCO_3_	20.2	22-46 mmol/L
White blood counts	4700	3.3-8.6x10^3^/μL
Hemoglobin	15.2	13.7-16.8 g/dL
Platelet	11.8	15.8-34.8 x 10^4^/μL
AST	187	13-30 U/L
ALT	51	10-42 U/L
γ-GTP	39	13-64 U/L
ALP	82	38-113 U/L
LD	710	124-222 U/L
Blood urea nitrogen	64	8-20 mg/dL
Creatinine	5.38	0.65-1.07 mg/dL
Creatinine kinase	13414	59-248 U/L
C-reactive protein	35.2	<0.14 mg/dL

**Figure 2:**
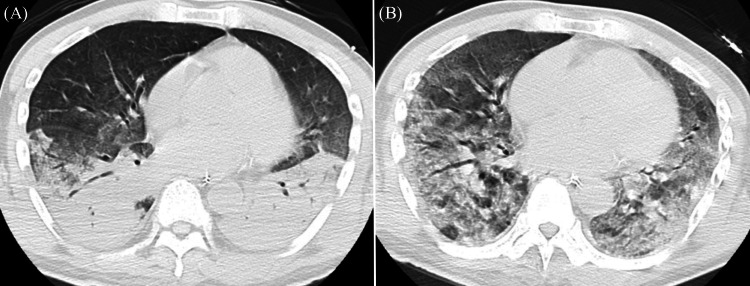
CT of the chest (A) On day 6, computed tomography (CT) of the chest showed bilateral alveolar infiltrates, suggesting the exudative stage of acute respiratory distress syndrome (ARDS). (B) On day 13, CT showed bilateral interstitial infiltrates, suggesting the proliferative stage of ARDS. CT, computed tomography; ARDS, acute respiratory distress syndrome

Immediately after admission to the intensive care unit, he presented with a temperature of more than 38°C, and his myoclonus persisted, suggesting serotonin syndrome. Eight hours after admission, his blood pressure dropped persistently despite administering norepinephrine and vasopressin. Cyproheptadine was not administered based on the limited evidence and theoretically worsen hyperthermia [5]. Thus, we performed esophagogastroduodenoscopy (EGD) to remove pharmacobezoars 10 hours after admission. EGD showed a large amount of pharmacobezoars in the stomach and about 10 tablets could be removed using a collection net (Figure 1C). However, the procedure could only be performed once due to a mild esophageal tear during the removal of the endoscope with a collection net and resistance at the pharyngoesophageal junction (Figure 1D). An oroenteric tube was retained for irrigation, and magnesium citrate was administered. Renal replacement therapy (RRT) was initiated on day 2, and RRT and administration of vasopressors were continued up to day 4. High fever and ocular flutter persisted up to day 7. Furthermore, lung oxygenation and lung compliance worsened daily despite administration of 3 g ampicillin/sulbactam, which was appropriate according to sputum culture results (Figure 2). Finally, severe ARDS developed, and 1 mg/kg/day of prednisolone was administered intravenously. However, his condition did not improve and he died of respiratory failure on day 23.

Examinations using a high-performance liquid chromatograph/tandem mass spectrometer (1260 infinity LC system and 6420 triple quad mass spectrometer (Agilent Technologies, Palo Alto, CA, USA)) revealed that serum concentrations of paroxetine were 5.38 µg/mL on admission and 3.21 µg/mL on day 7. Pharmacokinetic analysis of ingested drugs showed that only serum concentrations of paroxetine exceeded lethal levels for one week (Table 2) [6].

**Table 2:** Serum concentrations of ingested drugs during a period of one week after admission Serum concentrations were measured by a high-performance liquid chromatograph/tandem mass spectrometer (1260 infinity LC system and 6420 triple quad mass spectrometer). Therapeutic, toxic, and lethal levels were referenced by reference number 6.

Serum concentration (µg/mL)	Aripiprazole	Flunitrazepam	Lormatazepam	Paroxetine	Quetiapine	Trazodone	Zolpidem	Zopiclone
Day 0 (at admission)	1.42	0.15	0.37	5.38	1.32	2.21	0.42	0.14
Day 1	0.91	0.07	0.2	5.12	0.77	0.91	0.24	0.07
Day 2	0.74	0.03	0.09	3.64	0.34	0.54	0.09	0.02
Day 7	0.52	0	0	3.21	0	0	0	0
Therapeutic level (µg/mL)	0.1-0.35	0.005-0.015	0.002-0.01	0.002-0.065	0.1-0.5	0.7-1	0.08-0.16	0.055-0.085
Toxic level (µg/mL)	1	0.05	0.1	0.12-0.4	1-1.8	1.2-4	0.32-0.5	0.15-0.3
Lethal level (µg/mL)	1.9	0.11-0.74		1.2-4	0.95-12.7	9-15	1.5-4	0.6-1.8

## Discussion

A massive overdose of controlled-release paroxetine is likely to be fatal because the effects of paroxetine may last longer than expected. Thus, aggressive gastrointestinal decontamination may be recommended. Overdoses of SSRIs including paroxetine are common in cases of acute poisoning of prescribed drugs and usually have a good prognosis. Not all patients with an SSRI overdose need to visit an ED [1,7]. However, it may be necessary to conduct different management in cases such as our case with both persistent serotonin syndrome and the presence of a large amount of pharmacobezoars in the stomach. This assumption is supported by the unexpected clinical course and serum concentrations in our case. In our case, altered mental status, hyperthermia, and myoclonus due to serotonin syndrome continued for at least eight days. Initially, we did not expect this condition to be as severe and prolonged. However, the serum concentrations on day 1 (5.38 µg/mL) and even on day 7 (3.21 µg/mL) were higher than the lethal level (1.2-4 µg/mL) [6]. These high serum concentrations of paroxetine could lead to persistent serotonin toxicity. Reports of a lethal paroxetine overdose are extremely rare, but there has been one report of death due to pulmonary embolism on day 9 with an ingested dose of 4.5 g of Paxil CR® with serum concentrations on day 3 (3.42 µg/mL) and day 7 (4.42 µg/mL) [3]. Paroxetine controlled-release is well absorbed in the gastrointestinal tract and the plasma concentration reaches a peak concentration within six to 10 hours. The drug is widely distributed throughout the body and metabolized mainly in the liver by CYP2D6 enzymes. The elimination half-life of paroxetine controlled-release is 15 to 20 hours in a healthy population [3]. However, these pharmacokinetics are known to change significantly in cases of overdose. Considering this fact, prolonged high serum concentrations and serotonin syndrome can be partially explained by the high ingested dose. Moreover, controlled-release formulations may be significant contributors to prolonged high serum concentrations. Such formulations are prone to forming pharmacobezoars, resulting in the phenomenon [8]. Other possible reasons are considered to be the absorption of a residual drug and enterohepatic re-circulation, paroxetine metabolic characteristics as a non-linear dose-concentration relationship, and drug interactions with other concomitant drugs as in our case (e.g., quetiapine and trazodone) [9]. Our case finally led to death due to ARDS on day 23. The likely cause of ARDS was aspiration pneumonia. However, there have been some reports on the relationship between overdoses of SSRIs including paroxetine and ARDS, although the mechanism is not clear [10]. Given that the lethal serum concentrations of paroxetine persisted for a week, paroxetine toxicity might also be considered as a potential cause of ARDS. Further study is needed to determine whether a fatal paroxetine overdose causes pulmonary toxicity. Considering the unexpected clinical courses and pharmacokinetics, it should be recognized that a massive SSRI overdose is not as safe as it is generally believed, and the toxicity of the SSRI in a massive SSRI overdose may persist and lead to various complications including ARDS.

Two potentially effective treatments may be warranted in cases of massive SSRI overdose, including severe serotonin syndrome: gastrointestinal decontamination and a serotonin antagonist such as cyproheptadine. First, gastrointestinal decontamination for an SSRI overdose is not generally recommended due to lack of evidence, the possibility of complications, and the mild toxicity of SSRI [1]. However, considering the persistent toxicity of an SSRI overdose, gastrointestinal decontamination may be needed for severe cases such as cases requiring endotracheal intubation or high doses of vasopressors. Recently, there have been several case reports on the usefulness of endoscopic decontamination for critical overdoses such as potassium overdose [11]. Although the efficacy has not been established, it is theoretically possible to reduce the amount of absorbed drugs. As a result, both the peak serum concentration and the time above the upper limit of the lethal range may be reduced. In addition, EGD enables direct assessment of the residual amounts and drug formulations that cannot be identified by CT. Thus, consideration should be given to performing endoscopic decontamination in cases of ingestion of many tablets, many concomitant drugs, and prolonged-release formulations and in cases that are clinically severe [12]. However, there are still concerns about the safety of endoscopic decontamination. When a collection net is used to remove pharmacobezoars, the endoscope must be repeatedly moved in and out, which may cause esophageal and gastric injury as in our case. Endoscopic decontamination is an attractive option for the treatment of an overdose. However, further study is needed to establish the indications for endoscopic decontamination based on clinical severity and serum concentrations and to determine how to safely perform the procedure. Second, cyproheptadine, an oral antihistamine with some serotonin antagonist properties, has been considered for use in moderate to severe cases of serotonin syndrome [13]. However, cyproheptadine has not been associated with improved outcomes in critically ill patients with serotonin syndrome [5]. In addition, cyproheptadine could potentially exacerbate hyperthermia due to its anticholinergic properties. Because of these concerns, we refrained from administering this agent in our case. While there are medications such as olanzapine and risperidone that have anti-serotonergic activity, there is insufficient data to support their efficacy in the treatment of serotonin syndrome. The usefulness of serotonin antagonists in fatal cases of serotonin syndrome may be limited, and further research is warranted to determine whether outcomes are improved.

## Conclusions

An SSRI overdose is rarely life-threatening, and the treatment strategy in such a case has been well established. However, there is a lack of some pharmacokinetic information on an SSRI overdose including information on an acute toxic dose and maximum time to adverse drug reaction for each patient, especially in cases of a massive overdose of an SSRI. Clinicians should consider aggressive interventions such as endoscopic decontamination, especially in cases in which pharmacobezoars are present in the gastrointestinal tract. The detailed clinical course and results of pharmacokinetic analysis in patients with a massive overdose of an SSRI should be reported in the future to shed insight and further inform clinicians.
